# The Interplay between Viruses and Host DNA Sensors

**DOI:** 10.3390/v14040666

**Published:** 2022-03-23

**Authors:** Sandra Huérfano, Vojtech Šroller, Kateřina Bruštíková, Lenka Horníková, Jitka Forstová

**Affiliations:** Department of Genetics and Microbiology, Faculty of Science, Charles University, BIOCEV, 25250 Vestec, Czech Republic; vojtech.sroller@natur.cuni.cz (V.Š.); katerina.podolska@natur.cuni.cz (K.B.); lenka.hornikova@natur.cuni.cz (L.H.); jitka.forstova@natur.cuni.cz (J.F.)

**Keywords:** DNA sensing, innate immunity, DNA viruses, IFN, inflammasome, cGAS, IFI16, p204/Ifi-204, STING, TLR9

## Abstract

DNA virus infections are often lifelong and can cause serious diseases in their hosts. Their recognition by the sensors of the innate immune system represents the front line of host defence. Understanding the molecular mechanisms of innate immunity responses is an important prerequisite for the design of effective antivirotics. This review focuses on the present state of knowledge surrounding the mechanisms of viral DNA genome sensing and the main induced pathways of innate immunity responses. The studies that have been performed to date indicate that herpesviruses, adenoviruses, and polyomaviruses are sensed by various DNA sensors. In non-immune cells, STING pathways have been shown to be activated by cGAS, IFI16, DDX41, or DNA-PK. The activation of TLR9 has mainly been described in pDCs and in other immune cells. Importantly, studies on herpesviruses have unveiled novel participants (BRCA1, H2B, or DNA-PK) in the IFI16 sensing pathway. Polyomavirus studies have revealed that, in addition to viral DNA, micronuclei are released into the cytosol due to genotoxic stress. Papillomaviruses, HBV, and HIV have been shown to evade DNA sensing by sophisticated intracellular trafficking, unique cell tropism, and viral or cellular protein actions that prevent or block DNA sensing. Further research is required to fully understand the interplay between viruses and DNA sensors.

## 1. Introduction

During the last decade, various cell receptors that recognize pathogenic DNA and induce interferon, pro-inflammatory cytokine, and chemokine production have been discovered and have been termed DNA sensors. DNA sensors can be divided functionally into DNA sensors that mediate either interferon (IFN) type I (IFN α or β) or type III (IFN λ) and those that induce the activation of inflammasome-related cytokines, such as interleukin (IL)-1β or IL-18. The DNA sensors work as sentinels that recognize the DNA of intracellular microorganisms or mislocalized self-DNA (DNA leaked to the cytosol from the nucleus or mitochondria). Immune and non-immune cells are equipped with several DNA sensors, and to date at least 14 DNA sensor proteins have been described. Sensors can be classified, according to their subcellular location, as “cytosolic sensors” or endosomal receptors. Some “cytosolic sensors” have also been found in the nucleus; however, their role in the sensing of pathogen DNA or cellular damaged DNA in the nucleus remains controversial. In fact, several proteins that function as DNA sensors are multifunctional and play roles in other cell processes, such as the cell cycle or DNA damage responses. To date, the following DNA sensors have been identified: ZBP1(interferon-inducible protein—Z-DNA binding protein, also known as DAI (DNA-dependent activator of IFN-regulatory factors)), IFIX (interferon-inducible protein X), IFI16 (interferon gamma inducible protein 16), AIM2 (interferon-inducible protein 2) cGAS (cyclic GMP-AMP synthase), LRRF1P1 (leucine-rich repeat flightless-interacting protein 1), DDX41 (DEAD-box helicase 41), DHX36 (DEAH box protein 36), DHX9 (DEAH box protein 9), DNA-PK (DNA-dependent protein kinase), RNA pol III (RNA polymerase III), TLR9 (toll-like receptor 9), MRE11-Rad 50 (meiotic recombination 11 homolog-Rad 50), and Ku70 protein. After binding to DNA, the sensors engage adaptor proteins, such as the stimulator of interferon genes (STING), myeloid differentiation primary response 88 (MyD88), or β-catenin, which activate the interferon regulatory transcription factor (IRF)3, IRF7, and/or nuclear factor-κB (NF-κB). The transcription factors activate the promoters of the IFN type I or type III genes, cytokines, or chemokines. Model pathways are now emerging that show that sensors use specific adaptors and transmit signals to specific transcription factors. To date, STING has been found to be the adaptor most often used by DNA sensors. It is thought that the recognition of DNA by the sensors is in part redundant and DNA sequence independent (except for TLR9 and RNA pol III, which recognize un-methylated CpG motifs and dsDNA poly(dA-dT), respectively). Most DNA sensors are activated depending on their availability in the specific cell line and cell compartment (e.g., in endosomes, free in cytosol, or the nucleus). However, it is postulated that other features, such as length or conformation of DNA, may play a role in binding to these sensors. Given that most DNA sensors have only recently been discovered, aspects of the DNA recognition as well as pathways leading to cell responses are not fully understood. It is important to mention that, to date, the best understood DNA sensors are TLR9, AIM2, IFI16 and cGAS. The pathway mediated by cGAS-STING activation has been shown to be key to the cell response not only to viral infections, but also to cellular stress, tissue damage and cancer. This review will present an overview of the pathways of DNA sensing and their interplay with DNA viruses or viruses with intermediate DNA genomes. We will focus on the herpesviruses, adenoviruses, papillomaviruses, polyomaviruses, hepatitis B virus, and the retrovirus, human immunodeficiency virus (HIV).

## 2. Overview of the Innate Immune Sensors

The innate immune system involves immune and non-immune cells in all tissues. For innate immune sensing, cells use germline-encoded recognition receptors known as pathogen recognition receptors (PRRs). PRRs recognize specific pathogen molecular patterns (PAMPs) or, in certain circumstances, cell self-patterns known as damage-associated molecular patterns (DAMPs) produced by cell running stress responses. Specifically, the PAMPs can be lipids, proteins, lipopolysaccharides, or nucleic acids. Recognition via PRRs induces interferon or other cytokine production as a rapid response to fight invaders and to activate the adaptative immune response. PRRs can be divided into five families: (i) NOD-like receptors (NLRs); (ii) C-type lectin receptors (CLRs) that recognize lipids, lipoproteins, proteins, and glycans [1,2,3]; (iii) Toll-like receptors (TLRs) that recognize proteins, lipids, lipoproteins or nucleic acids [3,4]; (iv) Retinoic acid-inducible gene I–like receptors (RLRs) that sense RNA [5]; and (v) absent in melanoma 2-like receptors (ALRs) that sense DNA [6]. In addition, there is a group of sensors that has not been assigned to any family. The main sensors involved in nucleic acid sensing are members of the TLR family (namely TLR 3, 7, and 8, which sense RNA), TLR 9 (which senses DNA), members of the RLRs family RIG-1 (Antiviral innate immune response receptor RIG-1), also known as DDX58 (DEAD box protein 58), and IFIH1/MDA5 (Interferon-induced helicase C domain-containing protein 1/melanoma differentiation-associated protein 5) (which sense RNA), members of the ALR family (AIM2, IFIX, and IFI16 in humans/p204 (mouse ortholog), also known as interferon-activable protein 204, Ifi-204), which sense DNA [7,8,9,10,11]). Among the receptors that are not assigned to any family are cGAS, ZBP1/DAI, DDX41, DHX36, DHX9, DNA-PK, RNA pol III, MRE11-Rad 50, Ku70 protein and LRRFIP1. These sensors recognize DNA [12,13,14,15,16,17,18,19,20,21]. Most DNA sensors are multifunctional proteins that are also involved in other cellular processes, such as the regulation of transcription, cell cycle progression, apoptosis, and DNA damage response [22,23,24,25,26,27,28,29]. 

**Figure 1:**
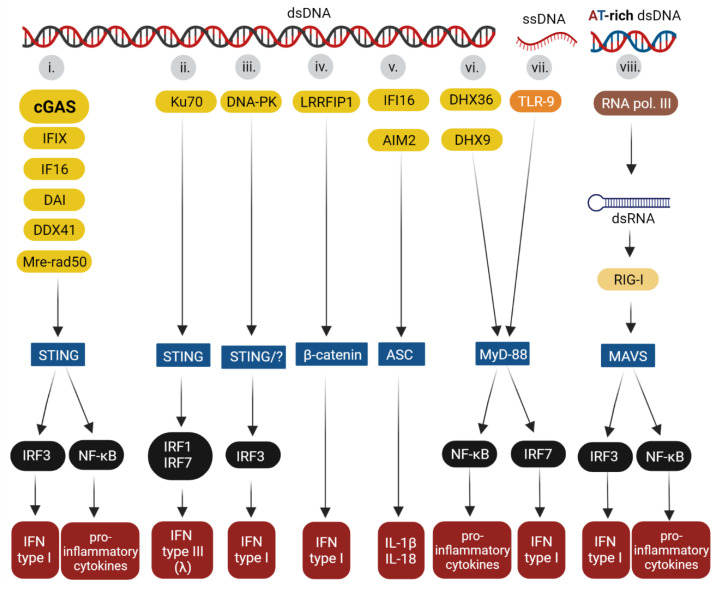
DNA sensors and their adaptor proteins involved in innate immune responses. The viral DNA that can be sensed by at least 14 pathways are: (i) dsDNA can be sensed by cGAS, IFI16, IFIX, ZBP1/DAI, DDX41, and MRE11-Rad 50 sensors that use the STING adaptor to induce the activation of IRF3 for IFN type I production and the activation of NF-κB for the production of pro-inflammatory cytokines; (ii) dsDNA sensing by Ku 70 protein activates IRF1 and IRF7 via STING for IFN-λ production; (iii) DNA/PK senses dsDNA via either STING or unknown adaptor activates IRF3 for type I IFN production; (iv) the sensing of dsDNA by LRRFIP1 via β-catenin to induce type I IFN production; (v) dsDNA sensing by AIM2 or by IFI16, resulting in the induction of inflammasome complex formation via ASC protein, leading to IL-1β and IL-18 production; (vi) DXH9 and DHX36 bind to dsDNA and engage the MyD88 adaptor, resulting in the activation of NF-κB or IRF7; (vii) TLR9 binds to ssDNA in endosomes and engages the MyD88 adaptor, resulting in the activation of NF-κB or IRF7, for the production of pro-inflammatory cytokines or IFN; and finally (viii) dsDNA enriched in AT is converted to AU dsRNA by RNA pol III. dsRNA is then sensed by RIG-1/DDX58 via MAVS adaptor protein to induce the production of IFN type I and other cytokines. Created with BioRender.com.

For each known DNA sensor, a specific pathway that leads to the production of IFN or other cytokines has begun to emerge. The adaptor proteins that are known to be activated by individual sensors are summarized in Figure 1. The protein STING is an adaptor activated by the following DNA sensors: cGAS, IFI16, IFIX, ZBP1/DAI, DDX41, and MRE11-Rad 50. Activated STING transmits signals to IRF3, which, after phosphorylation, translocates to the nucleus and induces the transcription of IFN type I genes [4,9,10,12,13,15,19,30]. The Ku 70 protein was found to activate IRF1 and IRF7, inducing the production of IFN-λ1 (Type-III IFN). Evidence for the role of STING as the adaptor protein for Ku 70 protein DNA sensors has been described in various cell lines [21,31]. The DNA-PK, which is a heterotrimeric complex (Ku70, Ku80, and a catalytic kinase subunit, DNA-PKcs), is involved in STING-dependent [20] and STING-independent [32] pathways that result in type I IFN production via IRF3. β-catenin is an adaptor used by LRRFIP1 (activated after the binding of DNA, but also RNA) [33]. After the ligand recognition, DNA (or RNA), LRRFIP1 recruits β-catenin. Consequently, β-catenin is phosphorylated and translocates to the nucleus where it binds to the IFN type I promoter. Other details of the pathway are unknown [33,34,35]. The adaptor for the proteins that induces inflammasome formation, AIM2 and, upon certain conditions, IFI16, is the apoptosis-associated speck-like protein containing CARD (ASC). The recruiting of ASC by the sensors induces the activation of caspase 1, which results in the production of IL-1β and IL-18 [6]. The sensors TLR9, DHX9, and DHX36 use the adaptor protein, MyD88, either by activating NF-κB for the production of inflammatory cytokines, such as IL-6 or IL-1, and tumor necrosis factor α (TNFα) or IRF7 for type I IFN production [16,36]. Finally, the RNA pol III senses AT-rich dsDNA and converts the dsDNA into AU-rich dsRNA, which is recognized by RIG-I/DDX58 (RNA sensor). Activated RIG-I/DDX58 uses as an adaptor the mitochondrial antiviral-signaling protein (MAVS), and their interaction leads to the activation of IRF3, IRF7, and NF-κB, resulting in the expression of type I IFN and other cytokines [18]. Below, the pathways activated by the most studied DNA sensors are described in more detail: (i) The TLR9 pathway that induces the production of proinflammatory cytokines and IFN, (ii) the AIM2 pathway as an example of signaling leading to inflammasome activation, (iii) IFI16-STING and (iv) cGAS-STING pathways inducing IFN production.

## 3. Molecular Bases of DNA Recognition

### 3.1. TLR9 Signaling Pathway

**Figure 2:**
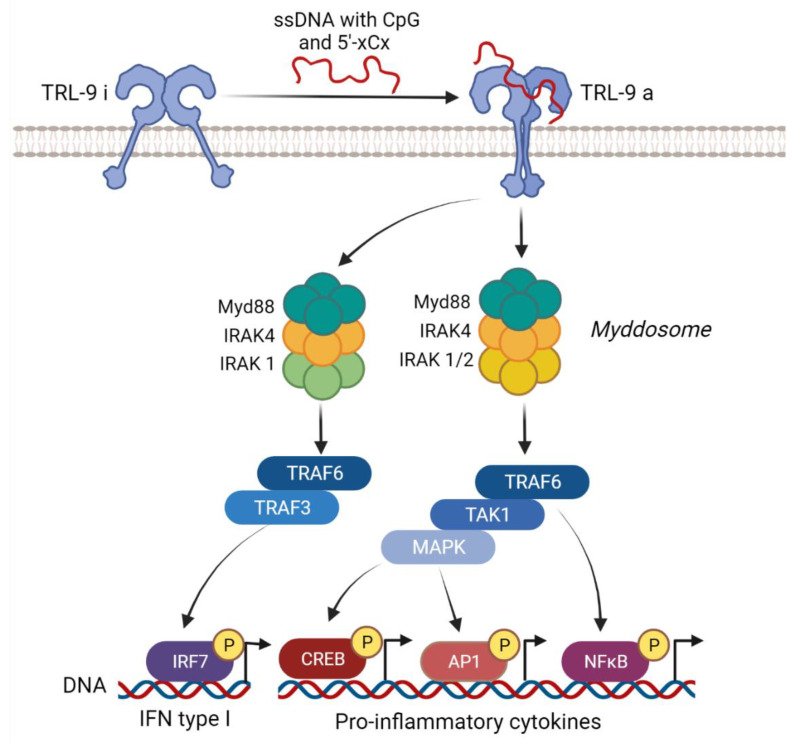
TRL9 pathway activation in response to DNA. TLR9 recognizes ssDNA enriched with CpG and 5′-xCx DNA motifs and changes the conformation of the TLR9 dimers. Dimers come into closer proximity and bind to the Myd88 adaptor. The adaptor recruits IRAK kinases and forms (i) myddosomes composed of MyD88, IRAK4, IRAK1, and IRAK2 that promote the sequential recruitment of TRAF6, TAK1, and MAPKs, leading to the activation of the transcription factors NF-κB, AP-1, and CREB for the production of inflammatory cytokines or alternatively (ii) myddosomes composed of MyD88, IRAK4, and IRAK1 promote the sequential recruitment of TRAF6 and TRAF3, leading to the activation of IRF7 and the production of IFN. Created with BioRender.com.

TLR9 was the first DNA sensor described in 2000 [36]. This sensor is abundantly expressed in B cells and plasmacytoid dendritic cells (pDCs) [37,38]. The protein exists as a dimeric homodimer and is composed of an extracellular domain with leucine rich repeat motifs, a transmembrane domain, and an intracellular TIR (Toll/IL-1 receptor (IL-1R)) domain [39]. TLR9 is localized in the endoplasmic reticulum (ER) and traffics through the classical secretory pathway to the endosomal system upon binding to DNA. TLR9 is first trafficked by the secretory pathway to the plasma membrane and then it is internalized by endocytosis mediated by the adaptor protein complex 2 (AP-2) [4,40,41]. Depending on the cell type and the appearance of TLR9 in different endosomes (early and late), the activation of different signaling cascades can be induced [42]. TLR9 recognizes single-stranded DNA with unmethylated cytosine–phosphate–guanosine (CpG) sequences [36,43] and it has recently been reported that it can also bind to DNA containing cytosine at the second position from the 5′ end (5′-xCx DNA) [36,44]. The TLR dimers are pre-assembled as a low-affinity complex before ligand binding. After binding ligands, dimers undergo conformational changes that bring the TLR molecules to more tight interaction, allowing for the reorganization of the TIR domains [45]. TIR domains recruit the MyD88 adaptor, which then recruits members of the dead domain (DD) containing serine/threonine IL-1R-associated kinases (IRAKs), IRAK4, IRAK1, and IRAK2, forming a signaling platform known as the myddosome. The myddosome is then associated with TRAF6 (the E3 ubiquitin ligase TNF receptor-associated factor), leading to the activation of TAK1 (transforming growth factor-β-associated kinase 1), which activates NF-κB. MAPK pathways are also induced downstream of the TAK1, causing the activation of the transcription factors activator protein-1 (AP-1) and CREB. As a result of the activation of the transcript factors, proinflammatory cytokines, such as IL6, TNFα, or chemokines, are produced [3,46,47,48]. In addition, in pDCs, dendritic cells (DCs), and macrophages, there is the production of IFNs upon the activation of TLR9 [49,50]. In this pathway, a myddosome formed by MyD88, IRAK4, and IRAK1 recruits TRAF6 and TRAF3, inducing the activation of IRF7. IRF7 induces the production of type I IFN [3,46,47,48]. The pathways activated during TLR9 signaling are summarized in Figure 2. 

### 3.2. The PYHIN Family Proteins IFI16, p204/Ifi-204 and AIM2: The Inflammasome and the IFN Signaling Pathways

**Figure 3:**
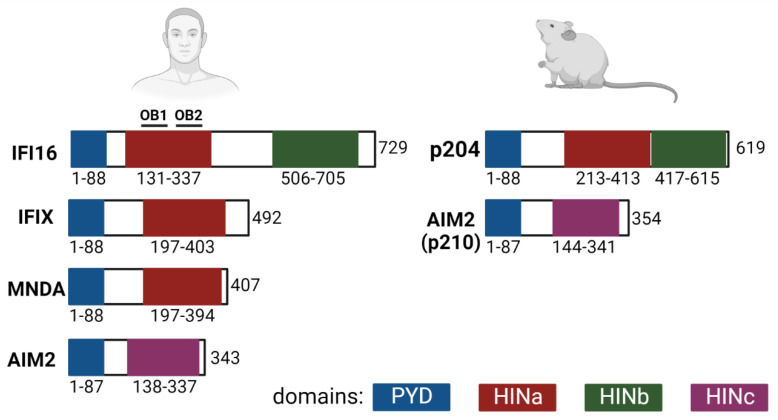
Domain organization of the human and mouse PYHIN family members. The PYD domain (blue), HIN domain a (red squares), HIN domain b (green), and HIN domain c (purple). The length of proteins and domains is presented. In the IFI16 HIN domain a, the position of the OBs is displayed. Created with BioRender.com.

The PYHIN family proteins are also known as hematopoietic interferon-inducible nuclear antigens with a 200-amino-acid repeat (HIN200). The family comprises 4 proteins in humans (AIM2, IFI16, PYHIN1, and MNDA) and 14 proteins in mice (Ifi-202 to Ifi-214). Human IFI16, IFIX, and AIM2 and mouse Ifi-210 (also known as AIM2) and Ifi-204 (also known as p204/Ifi-204) have been well characterized as players in immune responses. Mouse Ifi-210 is a direct ortholog of AIM2, while according to the phylogenetics tree of the pyrin domains, the human IFI16, IFIX, and MNDA form a clade with mouse p204/Ifi-204, p211/Ifi-211, p205/Ifi-205, and p207/Ifi-207 [51]. At present, p204/Ifi-204 is regarded as the mouse ortholog of IFI16 [52]. Although studies published in 2016 by Gray et al. [53] using PYHIN knockout mice (mice with depleted locus containing the PYHIN-encoding genes) showed that the proteins were indispensable for IFN responses, various studies have demonstrated that, in the mouse, p204/Ifi204 contributes to IFN responses induced by various stimulus [11,52,54,55]. Some of such studies are described in this review. In addition, examining the ALR locus in different inbred mouse strains, Nakaya et al. [54] found a high variability among them. Such differences could reflect the contrasting results obtained by Gray et al. [53]. PYHIN members have the N-terminal pyrin domain (PYD), which is a member of the larger superfamily of death domains and one or two C-terminal HIN (hematopoietic interferon-inducible nuclear) domains containing tandem pairs of oligonucleotide/oligosaccharide binding (OB) folds connected via a long linker with two α helices. The PYD domains allow the proteins to interact with the PYD domains of the same or other proteins to form homodimers or heterodimers. The HIN domains mediate DNA-binding in a non-sequence-specific manner that involves electrostatic interactions between the OB folds or OB fold linkers and the phosphate backbone of the DNA. HIN domains can be divided into three types (HINa, b and c). The specific features of some of the DNA sensors allow them to activate IFN responses or inflammasome responses. AIM2 induces inflammasome activation, IFI16 and its mouse ortholog p204/Ifi-204 can induce IFN type I or inflammasome responses [55,56] and IFIX has been shown to induce interferon responses [10]. An overview of the domain organization of the main PYHIN proteins is presented in Figure 3.

#### 3.2.1. AIM2 Inflammasome Signaling Pathway

**Figure 4:**
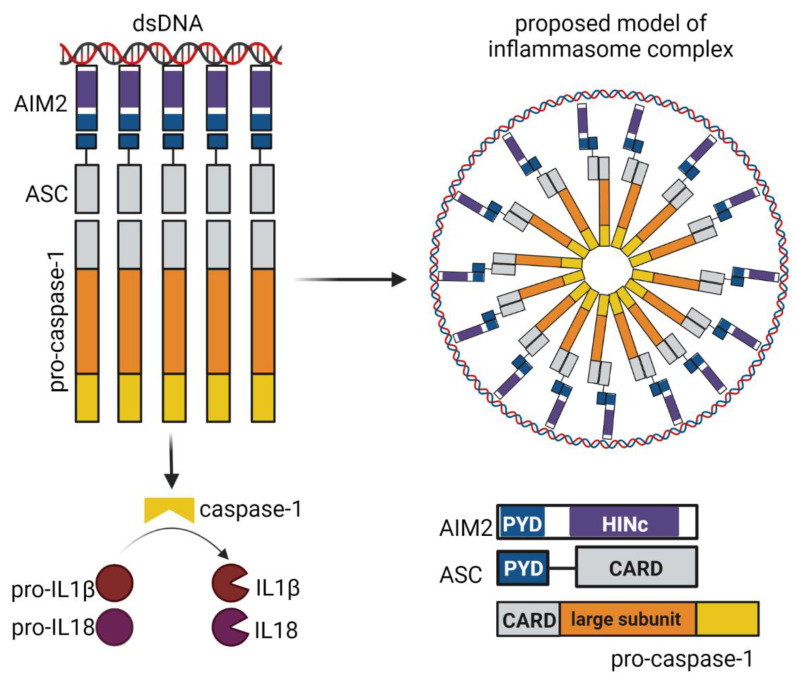
AIM2 inflammasome signaling pathway activation upon binding to DNA. AIM2 recognizes dsDNA and leads to AIM2 polymerization, then the ASC protein is recruited via PYD domains and forms filaments (nucleates). Caspase 1 binds to the dsDNA–AIM2–ASC complexes and is activated, leading to the maturation of IL-1β and IL-18. Created with BioRender.com.

AIM2 has one PYD and one HIN domain connected through a linker. AIM2 interacts with ASC protein via the PYD domain and binds to dsDNA via the HIN domain. The minimum length of DNA that induces the production of pro-inflammatory cytokines by inflammasome formation is 80 bp [57]. The HIN domain of AIM2 senses DNA by interaction with the OB folds. DNA–protein interactions involve the phosphate groups of the DNA and the side chains of positively charged amino acids of the HIN domain. For the regulation of the sensing, several lines of research have shown that, in the absence of DNA, the PYD domain is prevented from interacting with other proteins due to interactions with the HIN domain [58]. In contrast with previous results, it was shown that the AIM2 is not autoinhibited, but that binding to DNA and the size of DNA are crucial factors regulating oligomerization. The authors also suggest that, in the absence of stimulus, the AIM2 protein is at nanomolar concentrations and enough protein for polymerization/activation is only available during cellular stress when the level of the protein increases. Interestingly, it was also shown that the N-terminal PYD domain is required for the proper binding of DNA to the HIN domain [59]. The mechanism of AIM2 binding to DNA based on the above studies, together with other functional studies, have contributed to our understanding of the molecular events that lead to the production of pro-inflammatory cytokines via inflammasomes. In detail, after DNA binding, AIM2 recruits ASC adaptors and rapidly clusters into a seed filament, which is necessary to nucleate the polymerization of ASC, then recruits and activates procaspase 1, which causes the maturation of IL-1β and IL-18. Caspase-1 is recruited to the AIM2 inflammasome via the caspase activation and recruitment domain (CARD)’s homotypic interaction with ASC [59,60]. In a similar fashion, IFI16 and p204/Ifi-204 can induce inflammasome activation (see Figure 4).

#### 3.2.2. IFI16 and p204/Ifi-204 Binding to DNA and IFN Signaling Pathway

**Figure 5:**
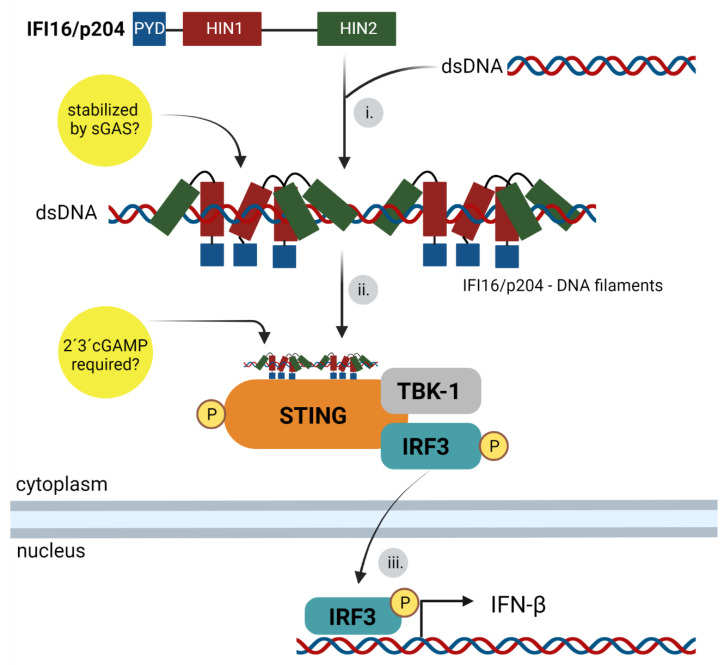
IFI16 or p204/Ifi-204 interferon signaling pathway. IFI16 or p204/Ifi-204 recognizes dsDNA via HIN domains, which induce HIN domains’ oligomerization, leading to the clustering of PYD domains. Next, the IFI16-DNA complexes travel to STING localized in perinuclear areas and then TBK1 and IRF3 are recruited. STING and IRF3 are phosphorylated by TBK1. Phosphorylated IRF3 translocates to the nucleus and activates the production of IFN β. The possible overlapping of the cGAS pathway with the IFI16-STING pathway is shown (the stabilization of IFI16 by cGAS or the requirement of 2′-3′ cGAMP for IFI16 STING stimulation). Created with BioRender.com.

The first mention of PYHINs as DNA sensors that induce IFN responses can be found in the studies of Unterholzner et al. (2010) [9]. They searched for novel DNA sensors that bind to the dsDNA by co-immunoprecipitation experiments and discovered the sensors IFI16 in human cell lines and p204/Ifi-204 in mouse cell lines. Moreover, their studies brought the first insights into the molecular mechanism of IFI16 binding and signaling. The analysis of the binding of DNA to HIN domains showed that the binding was stronger when both HINa and HINb domains were present. Authors also showed that IFN responses to transfected DNA 70 bp long were higher than responses to 60 bp DNA. On the other hand, base composition variations in the sequences of the 70 bp DNA did not result in any changes in the IFN responses. Additionally, they described that the pathway inducing interferon production is dependent on TBK1 and IRF3. Later studies revealed that the minimum length of the dsDNA for binding to the HINab domains is 16 bp for IFI16 and 20 bp for p204/Ifi-204 [9]. Nevertheless, it is important to point out that several studies have shown that the efficiency of IFN activation by the PYHIN sensors is related to the size of the DNA fragment. In fact, it has been reported that 150 bp DNA is favorable for interferon responses. Authors found that about ten IFI16 molecules comprise an optimal binding cluster (15 bp per IFI16) [56]. Based on the crystal structure of the individual or both domains bound to DNA, the molecular details of the binding of the HIN domains to DNA have been characterized. The HINa domain of IFI16 uses positively charged surface residues from the loop L45 of the canonical OB2 fold and the HINb uses charged residues from the linker region and the OB2 fold for binding to the DNA backbone [57,61]. Recently, details of p204/Ifi-204’s binding to DNA have also been described. HINa and HINb of p204/Ifi-204 bind dsDNA mainly through the α2 helix and the linker between them. In addition, it has been shown that the p204/Ifi-204 HINa domain mediates the dimerization of the protein [62]. Thus, for IFI16 and p204/Ifi-204, each HIN domain binds differently to the DNA, but both are essential for their function in IFN production. Insights into the connection of the ER resident adaptor protein STING with the IFI16 pathways came from different studies that showed STING to be essential for innate immune responses leading to the activation of IRF3 [9,63,64,65,66,67]. Other studies have proven that STING is required not only for the IFN responses to transfected DNA in various cell lines, such as macrophages, fibroblasts, or dendritic cells, but also for the in vivo responses of mice to herpesvirus simplex 1 (HSV-1) infection [68,69,70]. It has been shown that STING, in response to being triggered by DNA sensing, translocated from the ER to a perinuclear compartment that was identified as a non-ER microsome compartment. Additionally, authors have shown that brefeldin A blocked STING trafficking, indicating that STING translocated from the ER via Golgi to vesicles in the perinuclear areas. It has also been revealed that IFI16 and TBK1 colocalized with STING in the perinuclear vesicles. The vesicles with which STING, IFI16, and TBK1 were associated were positive for markers of early endosomes or for Sec5, a component of the exocytosis 8 subunit complex that facilitates vesicular transport and is associated with perinuclear endosomal compartments [64,65,68,71]. The details of the mechanism of IFI16 trafficking to STING is not well understood; however, a partial insight into IFI16 trafficking was gained during a study of IFI16 signaling in the nucleus during herpesvirus infection. The study showed that, after IFI16 binds to HSV-1 genomes, the IFI16 is acetylated. This modification has been shown to promote IFI16 trafficking towards cytosol [72]. Based on the above studies and on live imaging and electron microscopy that visualized the clustering of IFI16 on dsDNA [56,71,73] a model for IFI16 sensing could be unified. HINa and HINb domains of IFI16 or p204/Ifi-204 bind DNA synergistically regardless of its sequence (the only known restriction of binding is the presence of nucleosomes) and form a C-shaped ring-like structure around the DNA. Further, HINa dimerizes and brings PYD domains closer and IFI16 or p204/Ifi-204 complexes travel towards the STING compartments. Clusters of STING and IFI16 or p204/Ifi-204 recruit TBK1 and IRF3. TBK1 phosphorylates STING and IRF3 (more details of the STING activation are explained in the cGAS–STING pathway (below)). Phosphorylated IRF3 translocates to the nucleus and induces the transcription of type I IFN-β (Figure 5). Further, it was suggested that the IFI16 pathway is connected with the cGAS pathway. A study performed in human foreskin fibroblasts (HFFs) suggested that cGAS protein is required to stabilize the binding of the IFI16 to DNA in the nucleus under certain conditions. The authors found that cGAS interacted with the nuclear IFI16 during herpesvirus simplex 1 (HSV-1) infection, but not during DNA transfection. In addition, they observed that IFI16 has a shorter half-life when not in complex with cGAS [74]. cGAS was detected in a complex with IFI16 in human keratinocytes in response to DNA or viruses. The formation of such complexes is dependent on DNA stimulation. Moreover, the authors suggested that IFI16 synergizes the cGAS pathway since the dinucleotide synthesized after cGAS activation and the 2′-3′-cGAMP (2′-3′-cyclic guanosine monophosphate–adenosine monophosphate) interacts with IFI16 to activate STING. IFI16 knockout cells displayed lower IFN responses to 2′-3′-cGAMP-induced activation [75]. In macrophages, the depletion of IFI16 impaired 2′-3′-cGAMP production in response to dsDNA [76]. Taken together, these works indicate connections among signaling pathways, but the clarification of the crosstalk of the cGAS and IFI16 or p204/Ifi-204 pathways requires further study.

### 3.3. cGAS Binding to DNA and IFN Signaling Pathway

Initial studies described cGAS as a cytosolic sensor that detects pathogen DNA derived from viruses or bacteria and misplaced mitochondrial or nuclear self-DNA. cGAS catalyzes the production of the signaling dinucleotide, 2′-3′cGAMP, which as a second messenger activates the STING adapter molecule. STING connects DNA sensors and signaling molecules, such as TBK1 kinase and regulatory factor IRF3, which leads to the induction of type I IFNs [14,77].

Human cGAS consists of 522 amino acid (AA) residues. A positively charged N-terminus is disordered (1–160 AA) and plays a role in binding DNA and innate immunity activation, but also in other non-sensing functions or cGAS localization. The hyperphosphorylation of the N-terminus inhibits cGAS activity during mitosis [14,78,79,80]. C-terminus fragment (160–522 AA) contains a nucleotidyltransferase (NT) core domain (160–330 AA) with several conserved AA residues. Male abnormal 21 (Mab21) domain (213–513 AA) overlaps with NT domain and contains zinc-ribbon structural domain (390–404 AA), which determines the specificity of cGAS towards to dsDNA. Structural studies revealed three cGAS-DNA interfaces (sites A, B, and C) required for cGAS activation [81,82,83]. Compared to mouse cGAS, human cGAS exhibits reduced enzymatic activity due to substantial amino acid differences [84]. cGAS localizes not only in the cytoplasm, but also in the nucleus and at the cytoplasmic membrane of many cell types. The anchoring to the cytoplasmic membrane is mediated by the N-terminus of cGAS binding to membrane phospholipids [79]. The membrane localization of cGAS is important in distinguishing between endogenous and exogenous DNA, and consequently in the production of type I IFN in response to viral infection. The nuclear cGAS is tethered with intact chromatin and it has none or very low sensing activity.

DNA sensor cGAS is activated by binding dsDNA through extensive electrostatic interactions, hydrogen bonds, and attachment to the phosphate backbone of DNA [85]. The recognition of dsDNA is not sequence specific, but the length of the detected DNA is important. The minimum length of dsDNA activating cGAS is 20 bp; however, at this DNA length, cGAS activation is low [86]. Following dsDNA binding, cGAS forms dimers, stabilizing the enzymatic conformation of cGAMP synthase [85]. The formation of cGAS dimers rearranges the dsDNA into a high affinity form suitable for binding other cGAS dimers, which together form a ladder structure. By the process of liquid–liquid phase separation (LLPS), the cGAS–DNA complex condensates and forms droplet structures, which can be considered microreactors for the production of 2′-3′cGAMP [14,83,87]. Interestingly, recent work indicates that the pre-condensation of cGAS already occurs in the steady state due to binding protein G3BP1 (GTPase-activating protein-(SH3 domain) binding protein 1). Upon the accumulation of DNA in the cytoplasm, DNA actively displaces G3BP1 in cGAS–G3BP1 and condensates and promotes LLPS, leading to 2′-3′cGAMP production [88]. cGAS also binds dsRNA to form liquid droplets with dsRNA. However, this process does not induce a conformational change in cGAS [87].

Human STING consists of 379 AA residues. Four transmembrane domains are located at the N-terminus (21–136 AA). The cyclic dinucleotide-binding domain (153–340 AA) occupies the central area and protrudes into the cytoplasm. Part of the cyclic dinucleotide binding domain is a connector element (157–187 AA) that forms crossover points between two STING proteins. Disordered C-terminal tails (340–379 AA) contain phosphorylation sites for TANK binding kinase 1 (TBK1), which is essential for signaling [64].

**Figure 6:**
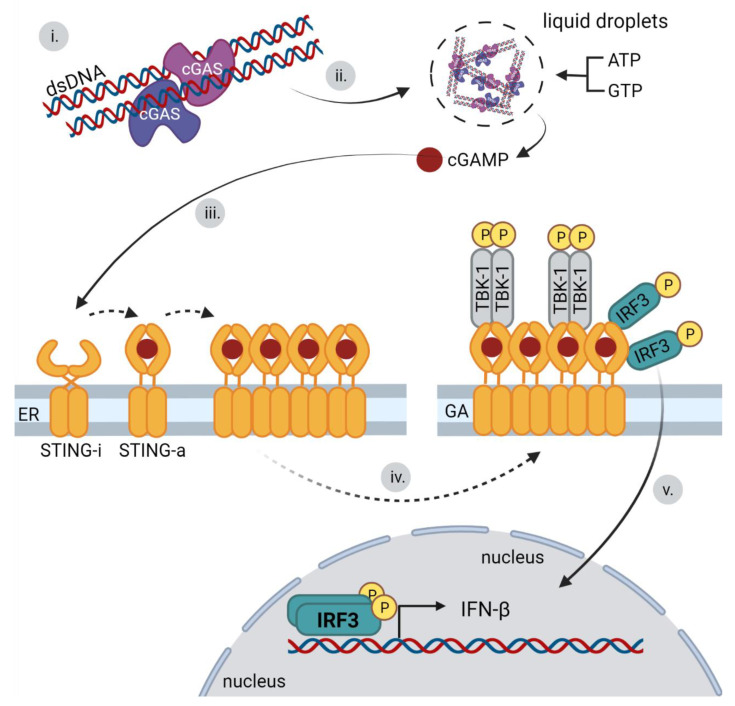
Crucial steps in the cGAS–STING pathway. (i) The binding of cGAS to dsDNA; (ii) cGAS dimers rearrange the dsDNA into a high affinity form suitable for binding other cGAS. The cGAS–DNA complex condensates and forms droplet structures where cGAS 2′-3′cGAMP is produced (from ATP and GTP); (iii) 2′-3′cGAMP binds to STING (STING inactive/STING-i), leading to its activation (STING-a). In detail, STING dimers oligomerize to linear tetramers; (iv) STING translocation from ER to GA via ERGIC. Sequential recruitment and phosphorylation of TBK1, STING, and IRF3; (v) Phosphorylated IRF3 enters the nucleus and activates the promotor of IFN-β.

STING is a transmembrane protein localized in a nonactive state at the ER as a dimer that is bound to TRAP β (translocon-associated protein β and translocon subunit SEC61β). The absence of TRAPβ and SEC61β impairs STING’s ability to induce the production of type I IFNs [77]. STING is retained in the ER through its binding to the Ca^2+^ sensor STIM1 [89]. STING is activated by the direct binding of cyclic dinucleotides (CDNs) mediated by a cyclic dinucleotide binding domain. Upon the binding of CDNs, STING dimers undergo a conformational change leading to its activation. The 2′-3′cGAMP ligand produced by cGAS has been identified as a STING activator [14]. Mutations in the STING cyclic dinucleotide binding domain lead to an inability to recognize CDNs and overall attenuation of the signaling pathway for the induction of type 1 interferons [90]. The cGAS–STING signaling pathway starts when cGAS sensors recognize and bind dsDNA. This leads to the synthesis of a second messenger, 2′-3′ cGAMP, by enzymatic activity of cGAS. This signaling molecule is subsequently detected by the C-terminal domain of STING, leading to its activation [90,91,92]. The STING activation consists of several consecutive steps. STING dimers first oligomerize to linear tetramers, which is made possible by the binding of cGAMP to the cyclic-dinucleotide-binding domain (CBD) of STING. Ligand binding to the CBD results in a 180° rotation of the CBD, which allows STING oligomerization. The conformation of inactivated STING does not allow for such a change. The mutation in CBD was shown to prevent STING translocation from the ER [93]. Simultaneously with the oligomerization of STING, its translocation from the ER to the ER–Golgi intermediate compartment (ERGIC) takes place. The binding of cGAMP to STING on the ER enhances the affinity of the STING protein for the SEC24C protein. The interaction with SEC24C, a component of the coat complex (COP-II), which promotes vesicular transport from the ER, leads to the trafficking of STING to the Golgi complex [94]. The next activation phase is the recruitment of TBK1 through the C-terminal TBK1-binding motif (TBM) of the STING protein, which is conserved across organisms. The TBK1 dimer, which interacts with two TBMs of dimerized STING, is not active and requires the phosphorylation of the serine residue. The activation of TBK1 requires its clustering, which leads to the closer contact of TBK1 dimers and subsequent trans-autophosphorylation. Because STING forms oligomers, it provides a suitable structure for TBK1 binding and oligomerization, leading not only to TBK1 autophosphorylation, but also to the phosphorylation of STING. Mutation in TBM preventing STING–TBK1 interaction does not trigger the IRF3-induced production of type I IFNs upon cGAMP stimulation. Phosphorylated STING recruits IRF3 and mediates its phosphorylation by the already bound TBK1 [66]. An earlier study showed that the C-terminus of IRF3 interacts with both the phosphorylated STING and already phosphorylated IRF3. As soon as the phosphorylation of STING-bound IRF3 occurs, unphosphorylated IRF3 is recruited and phosphorylated to form a dimer. IRF3 dimer separates from STING and enters the nucleus, where it induces the production of type I IFN. STING also activates NF-κB, which occurs prior to translocation of STING to the ERGIC [64]. Therefore, this activation is independent of TBK1 and IRF3, but the details of the activation are not clear [95]. The crucial steps of the cGAS-STING pathway activation are presented in Figure 6. In addition, STING ubiquitinylation at specific residues (e.g., K63 and K27-linked ubiquitination) have been shown to promote its activation [96,97,98]. The formation of STING-TBK1-2′-3′cGAMP condensates on the ER have been recently described as the mechanism preventing the overactivation of the cGAS–STING pathway, thus suppressing immune signaling [99]. In addition to interferon and proinflammatory cytokine production, autophagy activation is the important function of the cGAS–STING signaling pathway, which occurs after the infection of DNA viruses or DNA stimulation. The long-term presence of activated STING complex causes the continuous production or overproduction of IFNs. Such stimulation of the immune system leads to chronic diseases. STING-induced autophagy results in STING degradation in lysosomes [94].

In addition, 2′3′-cGAMP also induces LC3 lipidation in a way that is independent of TBK1 and the signaling C-terminal domain of the protein STING. The activation of autophagy by the cGAS–STING signaling pathway is thus independent of its ability to induce an IFN response. Autophagosomes then transport cytosolic endogenous or viral DNA together with activated STING for degradation in lysosomes [94].

## 4. Sensing of Viral Genomes by DNA Sensors

### 4.1. Immune Sensing of Human Herpesvirus Genomes

The *Herpesviridae* family consists of large, enveloped viruses of about 125–130 nm in diameter with linear double-stranded DNA genomes. Eight human herpesviruses have been identified. The herpesviruses most commonly used in immune sensing studies are herpes simplex virus (HSV-1), herpes simplex virus 2 (HSV-2), Epstein–Barr virus (EBV), human cytomegalovirus (HCMV), and Kaposi’s sarcoma herpes virus (KSHV). Herpesviruses encapsidate their 100–250 kbp long dsDNA genomes into capsids with icosahedral symmetry. The capsids are surrounded by a layer of tegument proteins that are enclosed within a lipid envelope. The herpesviruses attach to cellular receptors by envelop glycoproteins. They enter the cells via the fusion of the viral envelope with the plasma membrane or by endocytosis [100]. Released capsids travel toward the nucleopores and viral genomes reach the nucleus, where their transcription, replication, and packaging into capsids take place. Viral genomes are circularized and chromatinized after they enter the nucleus, but the density of the nucleosomes is low and their distribution irregular. In addition, histone associates with viral DNA less tightly than with the host DNA [101]. Apart from the lytic infectious cycle, herpesviruses can establish a latent infectious cycle. During latency, there is no production of viral particles due to the tight control of viral gene expression. Viral DNAs are stably maintained in the nucleus as episomes, which can be partitioned to daughter cells during division. For EBV, three latency programs have been described. Each program is characterized by different pattern expressions of the latency associated proteins [102,103].

Further, studies of herpesvirus genome sensing will be discussed and important contributions to the DNA sensor-mediated signaling pathways will be highlighted.

First, it was shown that HCMV, HSV-1, and HSV-2 are sensed by TLR9 receptors in pDCs. It was found that HCMV infects pDCs with low efficiency and that the cells are not permissive since only early products are expressed. The studies showed that the inhibition of the TLR9 pathway using a TLR9 antagonist reduced the levels of IFN-α secretion [104,105]. Additionally, studies on the sensing of HSV-2 by TLR 9 in pDCs revealed that an increase in endosomal pH by brefeldin A or chloroquine inhibited the production of IFN-α, thus highlighting the importance of intact endosomal pathways for TLR9 sensing. Moreover, it was demonstrated that in macrophages and splenocytesthe TLR9 sensor, sense genomes of HSV-1, eliciting the production of high levels of IFN [105,106,107,108]. Interestingly, the cytosolic sensor DHX9 (which uses Myd88 adaptor as TLR9) was found to be related to IFN responses in macrophages and fibroblasts infected with HSV-1. However, it was demonstrated that its action was not dependent on DNA sensing and the authors suggested that DHX9 functions in the cell nucleus as a co-activator of NF-κB and induces the production of antiviral cytokines [17].

Second, it has been reported that the sensing of genomes of KSHV, HSV-1, HCMV, and EBV by various DNA sensors, such as IFI16, cGAS, DDX41, or DNA-PK, resulted in the production of IFN-β or that sensing by IFI16 or AIM2 lead to inflammasome activation. Studies in HFF, macrophages and monocytes infected by HSV-1 have shown the involvement of IFI16 and cGAS in the IFN responses. It was shown that once the HSV-1 genomes localize in the nucleus, IFI16 binds immediately to them and induces the activation of IRF3, which results in IFN production [109]. In agreement with this, HSV-1 induced IFN production by STING activation in bone-marrow-derived macrophages (BMDMs) and silencing of IFI16 decreased the translocation of phosphorylated IRF3 to the nucleus and consequently reduced the levels of IFN-β production [9]. Other studies revealed that both cGAS and IFI16 are required for the IFN responses to herpesvirus infections. The authors used THP-1 cells (a human monocytic cell line) lacking IFI16, cGAS, or STING proteins and infected them with HSV-1 or CMV and found that the absence of cGAS or STING completely abolished the production of IFN β, while in the absence of IFI16, cells still produced low levels of IFN-β. The silencing of IFI16 in primary monocytes also resulted in low levels of IFN-β.

In agreement with the role of cGAS in the interferon responses to herpesvirus infection, mouse fibroblasts, macrophages, and dendritic cells lacking cGAS were found to be unable to launch IFN type I responses. Moreover, cGAS-knockout mice were more susceptible to HSV-1 challenge than wild-type mice [85].

The DNA sensor, DDX41, which interacts with and activates STING after DNA binding, has been shown to be directly involved in the sensing of HSV-1 DNA upon its transfection into myeloid dendritic cells [15]. Confirming the ability of DDX41 to activate the immune response to HSV-1 infection, it was shown that DDX41 mRNA is a direct target for miR-H2-3p of HSV-1. The expression of miR-H2-3p decreased the levels of DDX41, which resulted in a decrease in IFN-β production and attenuated antiviral response [110]. Another protein that has been shown to sense herpesvirus genomes is DNA-PK. One study demonstrated that knockout of DNA-PK in mouse cells launched attenuated IFN and cytokine responses to HSV-1 infection. The STING adaptor was shown to mediate the response since STING and IRF3 knockout cells did not initiate IFN responses [20]. A more recent study revealed an interaction between DNA-PK and IFI16. IFI16 was found to recruit DNA-PK to incoming HSV-1 genomes in the nucleus where the DNA-PK was shown to induce the phosphorylation of IFI16. The authors showed that the mutations of the phosphorylation sites of IFI16 resulted in attenuated IFN responses [111].Thus, the DNA-PK acts as a cofactor for IFI16 sensing.

Interestingly, some roles of STING in an antiviral response to HSV-1 not mediated by IFN were also suggested. It was found that the abolishment of IFN production in mice by substitution of STING serine 365 by alanine leads to mouse resistance to HSV-1 infection in a similar extend as exhibited by wild-type (WT) STING mice. In contrast, the knockout of STING affected negatively the responses of the mice to HSV-1. Thus, IFN-independent activities of STING mediate protection against HSV-1. STING-mediated autophagy is the additional mechanism (apart from IFN responses) that has been suggested to play a role in mice defense to HSV-1 infection [112,113].

Several studies with various herpesviruses and different cell lines showed that IFI16 can also sense viral DNA to induce inflammasome activation. The sensor IFI16 was found to bind genomes of KSHV in endothelial cells. Post-infection, complexes of IFI16, ASC, and caspase 1 were identified by co-immunoprecipitation. Confocal microscopy revealed that ASC and caspase 1 changed localization in the infected cells, where they colocalized in a speckled pattern in perinuclear space and in the nucleus. In contrast, both proteins displayed a diffuse pattern in non-infected cells. The knockdown of IFI16 or ASC, but not of AIM2, prevented the inflammasome activation [114]. In agreement with this, similar results were obtained for HSV-1 infected HFF cells. Viral genomes colocalized with IFI16 as early as 1 h post-infection and activation of caspase-1 was later detected (4 hpi). Co-immunoprecipitation experiments revealed an association between ASC and IFI16, but not AIM2 [115]. Inflammasome activation has also been observed in EBV latency cells I, II, and III (Raji cells, nasopharyngeal carcinoma cell line-NPC C666-1, and lymphoblastoid cell lines, respectively) [116].

Finally, a more complex picture of the sensing of the herpesviruses came from studies that simultaneously followed: (i) IFN responses activated by cGAS and IFI16; (ii) inflammasome activation; (iii) IFI16 trafficking; (iv) IFI16 post-translational modifications; and (v) the formation of IFI16 signaling complexes. These studies uncovered IFI16 acetylation during signaling and IFI16 interactions with BRCA1 (breast cancer gene 1) and H2B proteins. The first such study showed that IFI16 in the nucleus is acetylated by p300 acetylase during the sensing of KSHV (in human dermal microvascular endothelial cells (HMVEC-d) and HFF), HSV-1 (in HFF cells), and EBV (in primary human B cells and latency cells). The acetylation of IFI16 was shown to be required for the binding of IFI16 to ASC during inflammasome formation and also for IFI16-mediated STING activation. After acetylation, IFI16 translocated from the nucleus to the cytosol. In agreement with this, the inhibitor of acetylation, c-646, prevented the cytosolic translocation of IFI16, inflammasome formation, and the activation of the STING pathway [72]. Then, two studies found that during infection by HSV-1 (in HFF cells), KSHV (in human B lymphoma cells (BJAB- EBV-negative Burkitt’s lymphoma cell line)—or HMVEC-d) or EBV (in BJAB, latency and HMVEC-d cells), the IFI16 was bound to genomes in complex with H2B histone and with the BRCA1 protein. Additionally, it was shown that, while complexes of the three proteins were required for the signaling that induced IFN production, the absence of H2B did not affect the activation of inflammasomes. Intriguingly, the authors found that cGAS also interacted with IFI16 during the binding to STING [117,118]. The meaning of this interaction needs to be further clarified.

Inflammasome-mediated cytokine production and IFN production are mutually regulated. Thus, during infection by herpesvirus, the mutual regulation of the two types of responses determines the outcome of infection. Moreover, herpesviruses possess various mechanisms to downregulate both pathways. For HSV-1 infection, it has been shown that viral infected cell protein 0 (ICP0) immediately degrades IFI16, virion host shutoff protein UL41 downregulates the mRNA and protein level of cGAS, and tegument protein VP22 interacts with cGAS and directly inhibits its enzymatic activity [109,119,120]. The sensing of herpes virus is summarized in Table 1.

### 4.2. Adenoviruses Sensing by DNA Sensors

The members of the family *Adenoviridae* are nonenveloped viruses with icosahedral capsids with fibers (diameter about 80 nm). The genome is linear dsDNA of ~26–45 kb in length with two covalently linked terminal proteins. More than 100 different Human Adenovirus (HAdV) types are grouped into seven HAdV species A to G. Virus attaches to the primary receptor using fiber knob, is internalized by cells after the interaction of the capsid penton base with α-integrin, and then is released from the endosome to the cytosol. The capsid starts to disassemble while it is being transported along microtubules towards the nucleus. In the nucleus, the genome is transcribed, replicated, and the assembly of new virions occurs. The coxsackievirus-adenovirus receptor (CAR) is used by most adenoviruses as a primary receptor, and CD46 or desmoglein-2 is used by some adenovirus subgroups. Differences in receptor binding and the intracellular trafficking pathway affects the level of innate immunity activation [121].

The recognition of adenoviruses by innate immunity TLR9 sensor depends on the cell type [122,123,124,125]. Murine antigen presenting cells transduced by adenovirus produce type I IFN. In pDCs, TLR9 sensors and their adaptor, MyD88, were critical to the recognition of defective recombinant adenovirus (recAd) replication. Only WT pDCs produced IFN-α in response to transduced recAd, in contrast to TLR9-knockout pDCs. Conventional DC has been activated by recAd by both TLR9-dendent and -independent manners. TLR9 is also involved in IFN induction in the human THP-1 monocytic cell line or HeLa cells [126,127]. TLR9 was also required for NLRP3 inflammasome activation and production of IL-1β during adenovirus 5 infection of human macrophages [128].

Early interferon signaling in response to adenovirus infection has been studied in a murine macrophage-like cell line. cGAS protein was identified as a dominant cytosolic sensor for adenovirus as the knockdown of cGAS, STING, and TBK1 resulted in a dramatic reduction in the activation of IRF3 [129]. DDX41 sensors were also found to increase IRF3 activation in response to recAd in a murine macrophage cell line [130]. The RNA pol III transcribes small viral-associated RNA, which induces the production of type I IFNs in murine MEFs (mouse embryonal fibroblasts) and bone marrow-derived DCs [131]. The inhibition of RNA pol III by chemical components compromised late antiviral immune responses during adenovirus infection in a murine macrophage cell line. The authors also followed the role of ZBP1/DAI and p204/Ifi-204 in the IFN responses and found that the overexpression or knockout of ZBP1/DAI or p204/Ifi-204 had no impact in the IFN responses to adenovirus infection. Intriguingly, it was found that knockdown AIM2 cells displayed downregulated type I IFN signaling. The meaning of this finding needs to be further studied [130]. In Table 2, a summary of the DNA sensors that are responsible for IFN-mediated responses to adenovirus infection are presented.

Adenovirus immediate–early E1A protein interacts with many cellular proteins and blocks innate immunity by multiple mechanisms [132]. E1A transduced to the primary MEF cells has been found to inhibit the cGAS–STING pathway. The E1A LXCXE motif, essential to bind and inhibit protein pRb, is also critical to block the cGAS–STING pathway. Isoform E1A 12S and especially E1A 13S directly interact with STING through the LXCXE motif [133]. Downstream of the cGAS–STING signaling pathway, E1A, has been shown to interact directly with STAT1 and block STAT1-dependent transcription [134].

When WT- and E1A-deleted adenoviruses have been used for the infection of human HeLa cells, the cGAS–STING pathway activation levels were comparable at least up to 6 h post infection. The replication of WT adenovirus 2 did not seem to be affected by the lack of cGAS or STING in Hela or THP1 cells [129]. Thus, the effect of adenovirus evasion strategies is likely more pronounced downstream of the cGAS–STING signaling pathway.

### 4.3. Interplay between DNA Sensors and Human Papillomaviruses

Human papillomaviruses (HPVs) are small DNA tumor viruses that belong to the *Papillomaviridae* family. Papillomaviruses are diverse, and at least 450 genotypes have been identified [135]. They infect the epithelial cells of the skin and mucosa. According to the tumor potential, HPVs can be divided into low- and high-risk types. Low-risk HPVs cause benign lesions, while high-risk ones can induce premalignant or malignant processes and are causally related to human cancers, such as cervical or head and neck cancer. The virions of papillomaviruses are nonenveloped, formed by isometric capsids of icosahedral symmetry, 55 nm in diameter, contain circular dsDNA genomes approximately 8 kbps long, and found in complex with cellular histones. The papillomavirus genome contains a regulatory region and two early (E) and late (L) coding regions. The early region codes, according to the papillomavirus type, up to eight proteins (E1 to E8). From those, E1 and E2 are important for genome replication and transcription and E6 and E7 are viral oncoproteins. The late region codes for the capsid proteins, L1 and L2. Papillomaviruses replicate in the cell nucleus—their replication cycle is dependent upon the expression of the complete program of keratinocyte differentiation. The virus infects basal keratinocytes where only some E products are expressed. The subsequent steps in the life cycle depend on the differentiation process. Capsid protein L1 and L2 expression and morphogenesis of virions are restricted to differentiated keratinocytes [136]. Therefore, the propagation of HPVs in tissue culture is a challenge and, for a long time, only parts of the life cycle were studied in vitro. The papillomaviruses enter basal epithelial cells by endocytosis. In the endosomal pathway the virions disassemble and then L2/vDNA complexes (L2 complex of L2 capsid protein and viral genome) travel by retrograde transport to the trans-Golgi network [137]. The translocation of the complex to the cytoplasm has been suggested; however, the nuclear import of L2/vDNA complexes is thought to depend mainly on mitotic nuclear envelope breakdown [138].

A role for TLR9 in the sensing of HPV genomes has been suggested. TLR9 has been found to be overexpressed in a sub-set of samples from women carriers of cervix HPV. In fact, TLR9 levels were significantly higher in the low-risk HPV infected women and in persistently positive women [139]. Another study showed opposing results; the levels of TLR9 were found to be low in cell lines derived from cervical cancer (SiHa, CaSki and HeLa cells). TLR9 was found to be weakly expressed in SiHa and Hela cells and completely downregulated in CaSki cells. The authors hypothesized that E6 and E7 proteins could be responsible for the downregulation of TLR9. To prove this hypothesis, they overexpressed the E6 and E7 early proteins of HPV16 in human foreskin keratinocytes (HFK) or embryonic human primary keratinocytes and then challenged the cells with TLR 9 ligand (CpG ODN 2006). Cells displayed poor responses to TLR9 stimulation. The researchers found that the HPV16 proteins can inhibit the TLR9 promoter, E6 being slightly more efficient than E7. Thus, apparently HPV can modulate the TLR9 pathway [140].

During HPV infection, the induction of inflammasome response was detected. However, whether there is a contribution of IFI16 and/or AIM2 sensors in HPV genome recognition remains controversial. In skin biopsies from HPV16 positive patients, AIM2 was found to be more abundant than in controls. In addition, although IFI16 was equally abundant in the nucleus of HPV16 positive biopsies and controls, only in the HPV16 positive biopsies was a subpopulation of IFI16 found in the cytosol of the cells. The biopsies were positive for markers of inflammasome activation (IL-1β and cleaved caspase-1). Although the authors performed in vitro experiments to try to reveal the role of IFI16 or AIM2 in the inflammasome responses, the model used was the transfection of HSV genomes; therefore, the results do not account for papillomavirus infection, but rather for DNA transfection [141]. In another study, it was shown that human keratinocytes when infected with HPV16 virions, but not with HPV16 pseudovirions, produced IL-1β 4 h post-infection; however, the level of interleukin decreased rapidly. The authors showed that E6 and E7 viral early proteins block IL-1β production [142]. Thus, the rapid downregulation of inflammasome response and the difficulty of following the HPV life cycle in vitro are a challenge for the study of the role of sensors involved in the inflammasome responses to HPVs.

When searching for STING signaling in HPV16-infected HFK, no launching of IFN responses at the first 24 h post infection was detected [143]. Additionally, the authors did not observe any activation of IRF3 or STING. An analysis of the profile of transcription also did not show the upregulation of IFN-related genes. The authors hypothesized that HPV endosomal trafficking combined with the requirement of breaking down nuclear envelop allows the virus to efficiently escape from the immune system. To demonstrate this, experiments simulating the premature penetration of the endosomal membrane by HPV were performed. For this, cationic lipids were used to induce lysosomal escape of L2/vDNA complexes from endosomes. Under such conditions, an IFN response was detected, confirming the viral escape of HPV from the cytosolic sensing [143]. In addition, the negative regulation of STING was reported in cells derived from head and neck squamous cell carcinomas (HNSCCs) positive for HPV16. The stimulation of HPV16 positive or HPV16 negative HNSCCs by DNA transfection revealed that only HPV16 positive cells had a downregulated STING pathway and were unable to launch IFN responses. Moreover, IFN response to DNA was abolished by overexpressing HPV16 E7 in HPV16 negative HNSCCs. The authors also confirmed previous studies [133] that showed that the E7 protein of HPV18 interacts (via the LXCXE motif) with STING to inactivate it [144].

Table 3 briefly summarizes the downregulation of the immune sensing of HPV genomes by viral gene products.

### 4.4. Polyomaviruses Interactions with DNA Immune Sensing Pathways

Polyomaviruses (PyVs) are small nonenveloped dsDNA viruses that belong to the *Polyomaviridae* family. Their covalently closed circular dsDNA genome (approx. 5.3 kbp long) is in a complex with host cell histones (except H1) that are enclosed inside an icosahedral capsid that is ~50 nm in diameter. The regulatory region of PyV genomes containing replication origin, promoters, and enhancer separates early and late coding regions. The early region encodes early, so-called tumorigenic T antigens, multifunctional proteins that play a role in viral DNA replication, the regulation of virus and host gene expression, as well as in the modulation of the host cell immune responses and tumorigenesis. The late region encodes three capsid proteins—VP1, VP2, and VP3 [145,146,147]. Some primate polyomaviruses also encode agnoprotein, a regulatory protein whose function is not yet fully understood. Polyomaviruses are internalized by receptor-mediated endocytosis and travel via early and late endosomes to the ER. Then, they are released into the cytosol from where they translocate to the cell nucleus through nucleopores. In the nucleus, polyomaviruses use host cell functions for genome early and late transcription, alternative splicing, and genome replication. The release of new virions is connected with cell death, although an active virion release prior to cell death has also been observed [148,149,150,151].

Two human pathogens BK (BKPyV) and JC (JCPyV) polyomaviruses are known to cause serious illnesses in immunosuppressed patients [152,153] and human Merkel cell polyomavirus (MCPyV) is the etiological agent of 80% of Merkel cell carcinomas, an aggressive human skin tumor [154].

The polyomaviruses cause asymptomatic infections in their hosts and by a yet-unknown mechanism they establish viral persistence. The prevalence of human polyomaviruses (BKPyV, JCPyV, and MCPyV) could reach 40–90% in the adult population [155,156,157]. This suggests that polyomaviruses modulate immune responses to keep low levels of replication in infected hosts [146,158].

Although there are not many studies concerning the activation of TLR9 in polyomaviruses, it has been reported that in biopsies of patients with Merkel cell carcinoma there is decreased expression of TLR9 [159]. In agreement with this, it was shown that early antigens of MCPyV, BKPyV, and JCPyV are inhibitors of TLR9 [160]. In RPMI-8226 cells, the expression of early antigens of MCPyV, BKPyV, and JCPyV inhibited the activity of luciferase, whose expression was directed by the TLR9 promoter. Moreover, it was demonstrated that in epithelial cells the expression of the MCPyV–LT inhibited TLR9 expression by decreasing the mRNA levels of the C/EBPβ trans activator, a positive regulator of the TLR9 promoter [160].

Polyomaviruses induce type I IFN production in permissive host cells, as has been shown in cells infected with most studied human PVs, JCPyV and BKPyV, and model mouse polyomavirus (MPyV). Studies using MPyV and a recently developed possibility for studies of MCPyV using cells that allow us to follow the first part of the life cycle (entry, transcription and replication) have brought the first insights into the molecular pathways leading to IFN production in response to polyomavirus infection. The sensors cGAS and p204/Ifi-204 (mouse ortholog of IFI16) have been shown to be involved in the IFN response.

The study of Ryabchenko et al. [161] revealed that MPyV is apparently hidden from the innate immune system during its transport to the cell nucleus. However, the sensing of DNA occurred later, during viral genome replication in the nucleus, when STING and IRF3 became activated and IFN-β was upregulated. By using a mutant virus defective in nuclear entry, it was confirmed that in the absence of viral replication there is no launching of the interferon response. Thus, the presence of viral genomes in the nucleus is required for IFN-β induction via STING. Both p204//Ifi-204 and cGAS were shown to be involved in IFN induction. In the nucleus, p204//Ifi-204 colocalized with MPyV genomes and its downregulation led to a dramatic decrease in the IFN response. Additionally, the knockout of cGAS expression substantially hindered IFN production. Interestingly, cGAS, colocalized with cytosolic viral DNA, leaked from the nucleus, but also with MPyV genomes in the nucleus. However, after cell fractionation, the 2′3′-cGAMP dinucleotide was only detected in the cytosolic, but not in the nuclear fraction. Intriguingly, in the cytosol, co-localization of cGAS with micronucleus-like bodies whose presence increased with infection progression was observed. Thus, it is likely that viral DNA leaked from the nucleus to the cytosol and micronucleus-like bodies formed due to the genotoxic stress induced by the virus replication are sensed by cGAS. In parallel to the studies in MPyV, Krump et al. [162] performed studies in MCPyV using primary human dermal fibroblasts (HDFs) that support viral entry, gene expression, and replication [163]. The authors observed the upregulation of IFN response at late times post infection (around 140 hpi). At the same time, TBK1, IRF3, and NF-κB were activated and, moreover, 2′-3′-cGAMP production was detected. Using the knockout of individual proteins, STING, cGAS, IFI16, or TBK1, the authors confirmed that IFN production was induced by a cGAS–STING-dependent pathway. IFI16 was not found to influence the interferon response but, surprisingly, affected virus transcription. The mechanism of IFI16 in regulating the MCPyV infection in this model remains unsolved [162].

The absence of IFN induction at early times of PyV infection and the moderate interferon responses at late times post infection suggest that there exist various mechanisms that the PyVs use to evade immune sensing. It is evident that PyVs travel throughout the cytosol hidden in endosomes and their capsids are resistant to low endosomal pH. Although the virions partially disassembled in the ER translocate to cytosol prior to their importin-mediated transfer to the nucleus, their genomes tightly bound to histones and to VP1 are apparently not accessible for sensing. Moreover, possible released DNA could be rapidly degraded by exonuclease TREX-1. In the nucleus, incoming genomes seem not to be sensed or their sensing is below detection level as, regardless of multiplicity of infection, only a few genomes reach the nucleus [164].

Studies carried out with BKPyV have shown that, although the virus has a wide tropism (epithelial cells from lung, proximal tubule and medulla of kidney, bladder and urethra, fibroblasts from lung and bladder, and microvascular endothelial cells from lung and bladder), only the microvascular endothelial cells from the lung and the bladder were able to launch IFN responses. These results show that differences in the interferon response could direct the pathogenesis of PyVs. BKPyV infection of the renal tubular epithelial cells (RPTEs) is the cause of the kidney dysfunction and necrosis that result in renal fibrosis. Not surprisingly, the RPTEs cells are immunologically irresponsive to BKPyV infection [165,166,167] and are unlikely the sites of viral persistence. On the other hand, microvascular endothelial cells from the urinary or pulmonary tract that produce a low level of IFN are more likely to be the reservoir for the virus. IFN low levels could result in low levels of replication in the infected host [166].

For the RPTE cells, the mechanism of evasion of immune response have recently been described. Authors have shown that in infected cells at late times post infection (72 h), the agnoprotein targeted and disrupted the mitochondria and ER membranes. The disruption of mitochondria led to mitophagy. Authors have shown that the expression of agnoprotein in UTA6-2C9 cells treated with both poly(I:C)RNA and poly(dA:dT)DNA reduced the nuclear translocation of IRF3 [167]. Thus, agnoprotein affects nuclear sensing interfering with IFN production by affecting mitochondria. More details of the connection between mitochondrial damage and IFN responses need to be further elucidated and it should be clarified whether the binding of agnoprotein in the ER membrane can also cause the downregulation of STING pathways, e.g., by affecting STING interactions.

### 4.5. HBV Virus and the DNA Innate Immune Sensing Pathways

Hepatitis B virus (HBV) is an enveloped DNA virus approximately 42 nm in diameter classified into the *Hepadnaviridae* family. The HBV genome DNA is a relaxed-circular DNA (rcDNA) of approximately 3.2 kb in length with a complete minus strand and an incomplete plus strand. The viral genome encodes four overlapping open reading frames that code for three envelope S proteins (large, middle, and small), nucleocapsid protein (HBcAg), non-structural secreted protein (HBeAg), DNA polymerase (that has reverse transcriptase activity), and the multifunctional regulatory protein, HBxAg. HBV is a hepatotropic virus that recognizes one receptor for cell entry, the sodium taurocholate co-transporting peptide (NTCP), which is only present in hepatocytes. Additionally, the transcription of HBV genomes requires transcription factors enriched in hepatocytes [168]. After receptor binding, HBV virions are internalized by endocytosis [169,170]. The virus then reaches the cytosol and is imported into the nucleus with the help of importins. How the virus translocates from endosomes to the cytosol and where the disassembly of the nucleocapsids takes place is not clear yet. Once the rcDNA genomes appear in the nucleoplasm, they are repaired by host cell enzymes, resulting in the formation of covalently closed circular DNA (cccDNA). The genomic DNA then undergoes chromatinization [171] and is transcribed into mRNAs and pregenomic RNA (pgRNA), which are exported into the cytoplasm. In the cytoplasm, pgRNA is encapsidated and transcribed by viral reverse transcriptase into rcDNA inside the capsids. The nucleocapsids are either shuttled to the nucleus to amplify the pool of genomes for transcription or enveloped with host membranes containing S proteins to exit cells as the new HBV progeny.

HBV seems to be an inconspicuous virus for the innate immune system [172,173]. Its tissue tropism contributes greatly to this. A high level of exposure of the liver to microbial products and food-derived antigens from the intestines apparently provokes some kind of immunotolerance [174].

To date, there are controversial reports regarding the sensing of the HBV genomes or its DNA intermediates.

Although pDCs are important players in fighting viral diseases, various studies have shown that pDCs do not sense HBV by the TLR9 sensor [175,176,177] in spite of the efficient endosomal internalization of HBV virions in the cells [177]. Additionally, lymphocytes have been shown to escape viral sensing, as they do not secrete IFN α and IL-6 in response to HBV [176]. Various studies suggest that the cells actively block the TLR9 pathways. Wu et al. demonstrated that the presence of HBV virions inhibited the CpG-induced IFN-α production in PBMC [178]. Circulating and hepatic pDCs from patients with chronic HBV infection had defective responses to stimulation with TLR9 ligand [179]. Some mechanisms for TLR9 blockage/inactivation have been proposed, such as the downregulation of TLR9 expression, blockage of the MyD88-IRAK4 axis acting downstream of MyD88 and IRAK4 and upstream of IRF7 [176], or upregulation of SOCS-1 (suppressor of cytokine signaling 1) via HBsAg. [177].

The possible role of STING pathways and their related DNA sensors in the sensing of HBV has been explored. At first, it was found that IFN responses are not launched after the infection of hepatocytes by the HBV virus. For example, HepG2 cells overexpressing NTCP receptor infected with HBV of various multiplicities did not upregulate IFN or IFN-related gene production [180]. Additionally, studies of peripheral blood mononuclear cells (PBMCs) of patients with HBV (chronic or acute) showed no production of cytokine IFN-β or TNF-α [181]. It is important to mention that various studies have shown that the hepatocytes have low levels of DNA-sensing related genes and an absence of STING expression [182,183] or low levels of cGAS and STING [183]. Although in some studies hepatocytes have been shown to induce a moderate IFN response when HBV DNA or HBV genomes are introduced by transfection [182,183,184,185], there are controversial opinions concerning active intervention in the inhibition of DNA-sensing pathways by HBV. While the study of Lauterbach-Rivière concluded that in infected hepatocytes, HBV passively evades recognition by sensing its DNAs by cGAS/STING without active inhibition of the pathway [183], other studies have shown that infection by HBV suppresses components of the cGAS–STING pathway. The downregulation of the expression of STING, cGAS, and TBK1 genes in HepG2 cells infected by HBV was reported [180]. In addition, authors presented an in vivo experiment that showed that, although human hepatocytes in chimeric mice produced only low levels of cGAS, HBV infection resulted in a significant downregulation of the expression of cGAS and cGAS effector genes (including STING and TBK1) [180]. These results agree with previous studies that showed that hepatocytes downregulate STING production [182] or that monocytes from HBV patients produce lower IFN levels that controls in response to DNA transfection [181]. In another study, differentiated HepaRG cells and primary human hepatocytes infected or not infected with HBV were treated with cGAMP to compare IFN responses. Upon cGAMP stimulation, the IFN responses were significantly reduced in HBV-infected hepatocytes when compared with those in non-infected cells. The authors performed a functional screening by co-transfecting the constructs encoding HBV individual proteins together with the STING-expressing plasmid and IFN-β reporter plasmids into Huh7 cells. The screening showed that the viral polymerase (Pol) is responsible for the downregulation of STING. Other functional assays and co-immunoprecipitation experiments showed that Pol binds to STING and decreased STING-induced dimerization of IRF3 by inhibiting K63 polyubiquitination of STING [184]. Moreover, experiments showed that knockout of the STING pathway components (IFI16, TBK1, and STING) positively affected HBV infection [180].

Concerning the inflammasome response to HBV infection, it was shown that levels of IFI16 and AIM2 are higher in PBMCs of patients with acute or chronic hepatitis B and that at the same time production of inflammasome-related interleukins IL-1β and IL-18 were elevated in the serum of patients with acute hepatitis. However, the virus also downregulates this response. The authors presented evidence that HBeAg inhibits the activation of AIM2 and IFI16. The transfection of PBMCs isolated from chronic HBV patients with dsDNA induced the production of IL-1β and treatment with HBeAg after transfection abrogated the IL-1β expression [186]. It is important to mention that IFI16 has been shown to downregulate HBV infection by a method independent of the IFN or inflammasome response mechanism. It was shown that IFI16 binds to HBV genomes [187]. IFI16 binding to genomes promotes the epigenetic suppression of HBV cccDNA transcription by targeting an interferon-stimulated response element (ISRE) present in cccDNA (the HBV genome contains a typical ISRE). The study also revealed that HBV could downregulate the expression level of IFI16 in hepatocytes [188]. Table 4 summarizes the main mechanisms of the downregulation of innate immune sensing that have been described for HBV.

### 4.6. HIV and DNA Innate Immune Sensing Pathways

Human immunodeficiency virus (HIV) type 1 (HIV-1) and 2 (HIV-2) are members of the lentivirus genus of the *Retroviridae* family. HIV genomes consist of two copies of positive-sense single-stranded RNA 10 kb long containing encoding regions gag, pol, and env and genes for regulatory and accessory proteins. Gag codes for the structural proteins, capsid (CA), matrix (MA), and nucleocapsid (NC); pol encodes the enzymes reverse transcriptase (RT), protease (PR), and integrase (IN); and env encodes the glycoproteins gp120 and gp41. The regulatory genes are tat and rev, and the accessory products are protein Nef, Virion infectivity factor (Vif), protein Vpr, protein Vpu, and protein Vpx (Vpx is only present in HIV-2). Genomes are enclosed by capsid proteins, which forms a core with a conical shape. The core is covered by an envelope that contains host proteins and the viral glycoproteins gp41 and gp120. The HIV virions have a size of about 100 nm in diameter. HIV enters cells by a fusion process after binding to the CD4 receptor and chemokine coreceptors CCR5 and CXCR4 [189]. The viral lipid envelope fuses with the membrane of the host cell to deposit the viral capsid (CA) containing the RNA genome into the cytoplasm. Soon after entry, the reverse transcription of the genomes takes place, synthesizing a linear double-stranded cDNA version of the genome. There are conflicting reports regarding the uncoating of the virus: it was first believed that HIV capsids disassemble in the cytosol; however, it has been shown that the capsid is able to compact itself and enter the nucleopore [190,191]. In the nucleus, the provirus dsDNA is integrated into the chromosome. After integration, HIV-1 genes are transcribed by host RNA pol II with viral and cellular transcriptional cofactors. The translation of viral mRNA produces new viral proteins [191,192]. HIV-1 infections are distributed worldwide and correspond to more than 95% of HIV cases, while HIV-2 remains confined to West Africa. HIV-2 infected individuals are long-term non-progressors. In fact, the immune response to HIV-2 seems to be more protective, and therefore some of the studies mentioned below used both HIV types for a better understanding of the mechanism of modulation of the immune responses [193,194,195].

To date, only a few papers have reported the production of moderate levels of IFN or inflammasome-related cytokines in response to HIV infection, while various studies have demonstrated that the virus efficiently evades the innate immune system. In detail, Gao et al. showed that cGAS is implicated in the sensing of HIV cDNA. They found that the infection of THP-1 cells by HIV-1 led to the dimerization of IRF3 and production of IFN-β. Moreover, the production of 2′-3′cGAMP was detected in the infected cells. In agreement with this, the knockout of cGAS abrogated the IFN response. By using inhibitors of reverse transcriptase, the authors demonstrated the role of cDNA in cGAS activation, as cell treatment by azidothymidine or nevirapine resulted in the abolishment of the IFN responses. The authors proposed that cDNA and reverse transcription intermediates are sensed in the cytosol by cGAS [196]. Jakobsen et al. found that in addition to cDNA, the stem-rich ssDNA generated during the reverse transcription of HIV also contributes to IFN responses. Moreover, they showed that HIV-1 can be sensed by IFI16 sensor. In detail, the authors generated long synthetic ssDNA fragments of about 100 nt derived from three different areas of the 5′ UTR region of the HIV-1 genome and compared the ability of these fragments to stimulate IFN response in primary human monocyte-derived macrophages (hMDMs) and in THP-1 cells. Only stem-rich ssDNA triggered IFN responses, while the other linear short ss DNA forms did not. Further, using the knockdown of TPH-1 cells, the authors demonstrated that the IFN responses induced by transfected stem-rich DNA are dependent on IFI16, cGAS, STING, TBK1, and IRF3 [197]. It can be concluded that the cytosolic accumulation of viral DNA fragments (cDNA and ssDNA with stem-rich secondary structures) can be sensed by IFI16 and cGAS.

Studies on the mechanism of evasion of the innate immunity surveillance have often been carried out in DCs using HIV-1 and HIV-2 types. The reason is that DCs do not get efficiently infected by HIV-1 and do not induce the production of IFN, while HIV-2 can infect DCs and induce IFN production. The differences in the responses to the two HIV types are attributed to the interaction of the cellular enzyme SAMHD1 (SAM and HD domain containing deoxynucleoside triphosphate triphosphohydrolase 1) with HIV-2 Vpx protein. SAMHD1 depletes cellular pools of deoxynucleotide triphosphates, which results in the inhibition of the reverse transcription [198]. HIV-2 Vpx protein is responsible for targeting SAMHD1 for degradation [199,200,201].

Lahaye et al. created a HIV-2 mutant able to perform the reverse transcription of its genome, but the mutation prevents the delivery of cDNA to the nucleus. The generated mutant had a lower affinity for CypA protein, reported to modulate the capsid uncoating. Using the mutated virus, the authors revealed that the sensing of the reverse transcription products takes place in the cytosol by cGAS and does not require nuclear entry and integration of the DNA [202]. Additional studies confirmed the role of cGAS in the sensing of HIV DNA intermediates and brought new insights into the HIV evasion of sensing via GAS. The authors found that the protein NONO (DNA- and RNA-binding protein, involved in several nuclear processes) interacts with HIV capsids and promotes or drives the cGAS sensing of the HIV-2 cDNA. The NONO protein was found to bind both HIV-1 and HIV-2 capsids. However, the affinity of binding was substantially higher for HIV-2. Although the NONO protein was found not to be essential for the early phases of the replication of both HIV-1 and HIV-2, the sensing of HIV-2 by cGAS in DCs and macrophages was inhibited in NONO-depleted cells. NONO was found to colocalize with HIV-2 capsid protein in the nucleus and co-immunoprecipitation experiments showed that both NONO and cGAS are interacting partners. Thus NONO was found to be a capsid-binding factor that is essential for the cGAS-mediated recognition of HIV [203]. Next, it was shown that TREX1 DNA exonuclease is a restriction factor for HIV. A study demonstrated that infection by HIV-1 of hMDMs or CD4+ lymphocytes does not induce IFN production or results in a very modest IFN response (low levels of IFN after several days post-infection). However, when TREX1 was downregulated, the cells were able to launch a significant IFN response characterized by the production of IFN-β and IFN-α. The production of interferon was dependent on STING, TBK1, and IRF3 activation. In the TREX1 knockout cells, the cytosolic HIV cDNA was approximately 10-fold more abundant than in WT cells [204]. Another study, using TPH-1 cells, achieved similar results. TREX1 knockout cells have increased amounts of viral cDNA and upregulated the expression of IFN-related genes [205]. In agreement with this, using a humanized mouse model it was demonstrated that the knockout of TREX1 led to delays in viral replication by 3 to 4 weeks [206].

Finally, the HIV virus has also been shown to be sensed by IFI16 for the induction of inflammasome response. It was demonstrated that the majority of the quiescent CD4 T cells cultivated in human lymphoid aggregate cultures from tonsillar tissue when challenged with HIV-1 are not productively infected. In such cells, the accumulation of HIV DNA intermediate products in the cytosol, the activation of inflammasome response, and CD4 T-cell death by pyroptosis were observed. These resultsare a great contribution to the understanding of the mechanism by which HIV-1 kills CD4 T cells and suggested a possible role of DNA sensors (AIM2 or IFI16) in inflammasome formation during HIV infection [207]. Further studies have revealed the role of IFI16 in the inflammasome response during HIV infection. The knockdown of IFI16 in primary CD4 cells resulted in the inhibition of caspase-1 activation and decreased cell death; in contrast, the knockdown of AIM2 did not affect the inflammasome responses and did not prevent cell death [208]. A summary of the main proteins involved in the downregulation of DNA sensing in HIV infection are summarized in Table 5.

## 5. Perspectives

Although a great number of studies about the role of DNA sensors in innate immune responses to herpesvirus, adenovirus and polyomavirus have been carried out, it is apparent that there are some aspects that require further research to decipher the unknown molecular details of the DNA-sensing pathways. Various protein partners, such as BRCA1, H2B, and DNA-PK, that interact with IFI16 were discovered by studying the sensing of herpesvirus genomes in the nucleus. The interaction of IFI16 with those partners needs to be further studied in different contexts of DNA sensing to elucidate whether these interactions are general features of the IFI16 pathway or are exclusive for the sensing of herpesvirus genomes. Additionally, other questions should be addressed e.g., what is the role of the presence of IFI16 on almost fully chromatinized genomes of polyomaviruses in the nucleus? Additionally, to what extent can IFI16 function as a transcription repressor for polyomaviruses (as described for HBV)?

The mutual interactions of cGAS and IFI16 during herpesvirus sensing in the nucleus has been suggested. To date, the interactions have not been detected when sensing other DNA viruses [161]. DNA sensing in the nucleus by cGAS is controversial as some studies have failed to prove the activation of cGAS in the nucleus or have shown only low production of cGAMP during cGAS overexpression [78,161]. The deeper characterization of cGAS’ role in IFI16-mediated sensing of herpesvirus genomes needs to be carried out. Such studies will bring a greater understanding of the mechanisms by which cGAS manages to distinguish between self-DNA and invader DNA.

Unlike for herpesviruses, only a few studies have been carried out on adenoviruses. The sensors cGAS and DDX41 have been shown to play a role in sensing adenoviruses in non-immune cells. Since the knockout of cGAS dramatically decreases the levels of IFN, the cGAS is likely the predominant sensor in the recognition of adenoviral genomes. However, studies on the molecular steps that lead to the activation of and signaling by DDX41 during adenovirus infection would provide an insight into this pathway, which has not been yet fully characterized.

The sensing of micronuclei observed during polyomavirus infection has brought a great number of questions about the influence of the genotoxic stress induced by virus replication on the induction of IFN production. A new cytokine induction pathway with the participation of IFI16 and p53 and activated by DNA damage responses (DDRs), named the noncanonical STING activation pathway, has been reported [209]. Studies of the possible activation of this pathway by genotoxic stress induced by viruses replicating in the nucleus are desirable.

During polyomavirus infection genotoxic stress is induced (causing leakage of free DNA and micronucleus-like bodies into the cytosol), DDR is known to be activated by the early LT antigen [210,211,212], and the activation of cGAS and IFI16 has also been observed. Despite this, the levels of the innate immunity responses were moderate. Moreover, these viruses are capable of establishing persistent infection by limiting replication in some cell types. Therefore, polyomaviruses may be a good model for studying the modulation of IFN responses.

The studies that have been performed on papillomaviruses, HBV, and HIV discovered various mechanisms used by viruses to overcome innate immune sensing. These studies need to be further addressed as they are of great importance for rational target selection, including innate immunity components, for therapeutic intervention in DNA virus-caused diseases.

## Figures and Tables

**Table 1:** Important highlights in the innate immune sensing of herpesvirus genomes. Here, the DNA sensors that have been shown to play a role in herpes genomes sensing and the type of response they induce are presented. In addition, novel molecules or novel posttranslational modifications that have been found to play a role in the DNA sensing pathways are also shown. Dotted line (--) indicates absence of information.

Sensors	Type of Response	Novel Interacting Partners Identified for the DNA Sensing Pathways	Novel Posttranslational Modifications Identified for the DNA Sensing Pathways
TLR9	IFN [104,108]	--	--
IFI16	IFN	BRCA1, H2B [116,117,118]	IFI16 acetylation [73,118,119]
cGAS	IFN	IFI16 [76]	--
IFI16	Inflammasome	H2B [118]	IFI16 acetylation [118]
DDX41	IFN [15,110]	--	---
DNA-PK	IFN	IFI16 [20,111]	IFI16 acetylation [20,111]

**Table 2:** DNA sensors activated during adenoviral infection and the cell types in which they have been described.

DNA Sensors	Cell Types
TLR9	pDCs [122,123,124,125] Monocytes-TPH-1 [126] Epithelial cells-HeLa [127]
cGAS	Macrophages [129]
DDX41	Macrophages [130]
RNA Pol III	Fibroblast-MEFs [131] DCs [131]

**Table 3:** Viral proteins and events that lead to the downregulation of the immune sensing of HPVs.

	TLR9	Inflammasome-Related Pathway	STING-Related Pathways
Mechanism of down regulation	HPV16 E6 and E7 downregulate TLR9 expression [140]	HPV16 E6 and E7 block the IL-1β production [142]	Viral entry prevents genome exposition in cytosol [143] HPV18 E7 interacts with STING preventing its activation [133,144]

**Table 4:** Viral proteins and events that lead to the downregulation of innate immune sensing of HBV.

Pathways	Inflammasome Related Pathways	STING-Related Pathways
Mechanism of down regulation	HBeAg blocks IL-1β expression [186]	Hepatocytes have low levels of STING and cGAS [180,182,183]. Hepatocytes infected by HBV have down regulated cGAS, STING and TBK1 levels [180]. HBV pol interacts with STING preventing polyubiquitination (required for activation) [184].

**Table 5:** Host and viral proteins and events that lead to the modulation of the innate immune sensing of HBV.

Proteins	Function	Influence on the DNA Sensing Response
SAMHD1	Depletes cellular pools of deoxynucleotide triphosphates	Down regulate DNA immune sensing by affecting the availability of deoxynucleotides for reverse transcription [198,201]
TREX1	DNA exonuclease	Down regulate immune sensing by degrading HIVDNA intermediates [204,205,206]
NONO	Binding to capsids	Promoting sensing by cGAS by unknow mechanism [203]
Vpx	Accessory protein	Targets SAMHD1 for degradation [199,200,201]

## Data Availability

Not applicable.
